# Comorbid symptoms of depression and musculoskeletal pain and risk of long term sickness absence

**DOI:** 10.1186/s12889-018-5740-y

**Published:** 2018-08-06

**Authors:** Ole Melkevik, Thomas Clausen, Jacob Pedersen, Anne Helene Garde, Andreas Holtermann, Reiner Rugulies

**Affiliations:** 1 NRCWE, Copenhagen, Denmark; 2 Department of Sports Science and Clinical Biomechanics, University of Southern Denmark, Odense, Denmark; 3 Department of Public Health, University of Copenhagen, Copenhagen, Denmark; 4 Department of Psychology, University of Copenhagen, Copenhagen, Denmark

## Abstract

**Background:**

Symptoms of depression and musculoskeletal pain have both been found to be associated with increased risk of long term sickness absence (LTSA). The comorbidity between depression and pain i.e. simultaneous presence of both symptoms, is well established in the literature. The aim for the current investigation was to investigate whether the presence of comorbid pain influences the associations between depressive symptoms and LTSA or if the presence of comorbid depressive symptoms influences associations between musculoskeletal pain and LTSA.

**Methods:**

A sample of 6572 Danish female health care workers responding to a questionnaire about health and working conditions were followed up in a national register of social transfer payments (DREAM) for 550 days. We estimated the risk for LTSA of four weeks or more, associated with depressive symptoms and number of musculoskeletal pain locations using a Cox proportional hazards model allowing multiple observations per individual. We conducted a test for multiplicative interaction between musculoskeletal pain locations and depressive symptoms, and presented stratified regression models to facilitate the interpretation of the results.

**Results:**

The severity of depressive symptoms was correlated with the number of pain locations reported (Spearman’s rho = .24, *p* < 0.001). We found a significant multiplicative interaction between depressive symptoms and musculoskeletal pain in predicting the risk of LTSA. Depressive symptoms and number of musculoskeletal pain locations were associated with increased risk of LTSA for individuals who did not have comorbid symptoms. However, we found no significant associations between the two predictors and LTSA among participants who reported comorbid symptoms.

**Conclusions:**

The risk of LTSA associated with depressive symptoms and musculoskeletal pain appears to be moderated by the presence of comorbid symptoms. The modified risk for LTSA among workers with comorbid symptoms requires further investigation.

## Background

Depression and musculoskeletal pain are two well known risk factors for long term sickness absence (LTSA) i.e. sickness absence of several weeks [1–5]. Depression is the fourth leading cause of disability worldwide [6] and has been found to be associated with increased risk of LTSA, with some studies showing increased risk even at sub-clinical levels [1, 4, 7]. Musculoskeletal pain, including neck/shoulder pain, knee pain and lower back pain, has been found to be independent risk factors for LTSA [2], and there is also evidence suggesting that widespread pain [5], or the number of pain sites [8] are associated with increased risk factors for LTSA.

The understanding of how depression and pain may lead to LTSA is complicated by the tendency of pain and depression to cluster within the same individuals. A review by Bair et al. [9] reported that the level of comorbidity varies substantially according to the study population and the measurements used. On average 65% of patients with depression experience one or more pain complaints, ranging from 15 to 100%. Whereas, the prevalence of patients with pain conditions, who also experience major depression has been found to vary between 5 and 85% [9].

To our knowledge, no studies have yet specifically addressed whether the risk of LTSA from depressive symptoms and musculoskeletal pain remains consistent in the presence of comorbid symptoms. Thus, it is unclear if the well-known comorbidity between pain and depression [9] might be a cause of bias in previous studies reporting on how depressive symptoms and musculoskeletal pain are associated with LTSA in previous studies [1, 3, 4, 7].

Despite the lack of directly comparable studies, there are two studies which have results which may be relevant to compare our results to. In a population of older Americans, Emptage and colleagues [10] found that increasing severity in pain was a stronger predictor of changes in employment status and onset of work disability relative to depression. Having simultaneous symptoms of pain and depression was not found to be associated with significantly different outcomes compared to pain level alone. Demyttenaere and colleagues [11] utilized a cross sectional study with retrospective accounts of working days, quality and quantity of work performance because of problems with physical health or mental health or use of alcohol or drugs (the work loss index of the world health organization). They found that a combination of major depressive episode and high levels of painful physical symptoms were associated with a higher work loss score relative to each disorder alone, but that there was no interaction between the two risk factors.

The lack of knowledge of whether symptoms of depression and pain interact in predicting the risk of LTSA has practical implications for the design of interventions aimed to reduce LTSA. Specifically, if the risk associated with each risk factor changes (either is amplified or attenuated) with the presence of comorbid symptoms, then this could imply that specific subgroups are at particularly high risk, or that comorbid symptoms do not at all change the risk for LTSA for someone who already has one risk factor.

In light of the reviewed literature, we expect to find that pain and depressive symptoms cluster among Danish female paid care workers. Further, expect to find that crude models will indicate that both depressive symptoms and musculoskeletal pain is significantly associated with LTSA. Finally, we aim to test whether the risk of LTSA associated with musculoskeletal pain and depressive symptoms respectively, is consistent across levels of the other risk factor. This test will be explorative, as we do not have specific expectations regarding the potential interaction. For the purpose of this investigation, we utilized a cohort of female paid care workers. This group has previously been found to be vulnerable to musculoskeletal pain, depressive symptoms and LTSA [2, 12] and may therefore be suitable for our investigation.

## Methods

### Sample

This study uses survey-data collected among employees in the elder-care services in 10 Danish municipalities in 2008–9 linked to a national register on social transfer payments (the DREAM registry) [13]. The survey was the third wave of a prospective cohort study. Paper-and-pencil questionnaires were sent to 13,383 employees and the survey yielded a response rate of 63.0% (*n* = 8431). In the analyses we first excluded participants who were not engaged in the direct provision of care services (*N* = 1655) and then excluded male respondents (*N* = 204), yielding a final sample of 6572 respondents. If the response rate is calculated based upon women who worked in care-providing jobs in 2006, the response-rate is 60%.

Respondents were followed in the DREAM register for 550 days after completion of the survey. DREAM contains weekly information on granted sickness absence compensation for all citizens and residents in Denmark.

When comparing distributions of depressive symptoms, musculoskeletal pain.

age, and job-group from the survey in 2006, we found no sign of systematic differences between responders and non-responders of the current sample.

### Outcome

LTSA was operationalized as any sickness-absence for four consecutive weeks or more during the 550 days following the response to the questionnaire. LTSA was measured in the DREAM register and this absence period was chosen as it was the shortest possible absence period we could measure in the DREAM register. Respondents who were on sickness absence at the time of the baseline survey were excluded from the regression models (*N* = 164) leaving a final sample of 6408 participants. Sickness absence periods that started during follow-up and continued after the end of the follow-up period were included. Respondents who died, retired or emigrated during follow-up were censored from the analysis at the time of death, retirement or emigration. Participants were allowed to contribute with multiple time periods under risk if they had spells of unemployment, or if they for any other reason fell temporarily out of the DREAM registry during time for follow-up.

### Predictors

Participants were asked to rate the average levels of musculoskeletal pain in low back, neck/shoulders and knees over the last three months. The response scale ranged from 0 to 9 where 0 was “no pain” and 9 was “worst imaginable pain”. A drawing from the Nordic Questionnaire defined the three respective body part regions [14]. We used a cutoff of 3 points on each scale to indicate whether participants had substantial levels of pain. This decision is based upon the work of Andersen and colleagues [2] where this same instrument was used and the risk of LTSA was found to increase at 3 points for pain in the knees, 4 in the /neck shoulders and at 5 in the lower back. Next, we constructed a count variable indicating the number of pain locations experienced by each participant. This ranged from 0 (no pain) to 3 (pain in all three assessed body parts). When used as an independent variable in the regression models, this variable was used as dummy variables with 0 pain locations as a reference value.

Depression was assessed by the Major Depression Inventory (MDI) [15]. This scale includes 10 items reflecting depressive symptoms on a scale from 0 (no symptom experienced) to 5 (experiencing the symptom all the time). We included all respondents who answered 5 or more questions of this scale and generated a mean score which we multiplied by 10. Thus, the final depression scale had a theoretical range between 0 and 50. We generated a variable with three levels of depressive symptoms (low: from 0 to 12.99; moderate: from 13 to 20.99, high: from 21 and more). These categories correspond with the categories suggested in Olsen et al. [16] where the range of low depressive symptoms are described as *no depression/full remission*, the medium symptom level is described as *probable major depression/mild depression* and the high symptom level is described as *major depression/moderate depression*. The low category serves as a reference group representing levels of depressive symptoms which are not likely to be associated with elevated risk of LTSA. For more in-depth description of the MDI scale, see Bech et al. [15].

### Covariates

We used a set of covariates to adjust analyses for more precise prediction of risk and to avoid potential confounding. Age (in years) [17, 18] and cohabitation status (yes, no) [18, 19] were associated with LTSA in previous studies. Job-title was also included (as dummy variables) as risk for LTSA has been found to vary across job-groups [20]. The job-titles included were healthcare assistants, nursing aids, nurses and therapists, and other care staff. Finally, seniority at current employer (in years) was included as sickness absence has been found to be less common among individuals with longer seniority [21].

The sensitivity analyses also included controls for smoking (current smoker, or not), leisure time physical activity (four levels included as dummy variables) and physical work demands (a continuous measure assessing the cumulative physical work from very, very light to very, very strenuous).

### Statistical analysis

We used person chi-square tests and Spearman’s correlation coefficient to assess whether symptoms of depression and the number of pain locations are independent from each other, or if the symptoms tend to cluster within individuals. We used Cox proportional hazards models to test for the associations between depression, pain and LTSA. The time scale utilized counted from the day the questionnaire was completed until the date when an individual was registered with LTSA in the DREAM registry, was censored due to death, retirement or immigration or end of follow-up), whichever came first. We estimated crude models for each risk factor, mutually adjusted models (Model 1) and a mutually adjusted model including covariates (Model 2). Interactions between pain and depression (Model 3) were tested by including main effects for both number of pain locations and depression while adding multiplicative interaction terms for the effect of pain with low, medium or high levels of depression. The interaction models were tested with statistical adjustment of age, seniority and cohabitation.

We also performed sensitivity analyses for model 2 and model 3 including additional control variables including smoking (current smoker, or not), leisure time physical activity (four levels included as dummy variables) and physical work demands (a continuous measure assessing the cumulative physical work from very, very light to very, very strenuous). These variables could potentially confound the associations between depression, pain and LTSA, but were not included in the main analyses as they were measured at the same time as the independent variables, and could therefore potentially both confound and/or mediate the associations of interest.

The statistical significance of the interactions was tested using adjusted Wald tests that tests whether all multiplicative interaction terms are equal to zero. In case of significant interactions, we proceeded to explore the survival models in stratified models. The survival models were done using the Breslow method for ties and robust standard errors to account for multiple observations within some participants. Incidence rates were presented as the number of cases per 1,000,000 person days. Interactions were interpreted by means of regression models stratified by both depressive symptoms and the number of pain locations. All statistical analyses were performed in STATA/SE (version 15) [22].

## Results

The mean age of the participants was 45.9 years (*SD* = 10.5), the mean level of seniority at the current employer was 8.3 years (*SD* = 8.2), and 79.2% of the sample were cohabiting.

Table 1 shows the distribution of symptoms between individuals with low, medium and high levels of depressive symptoms across the number of pain locations. The distribution of depressive symptoms showed that about 80, 13 and 7% reported low medium and high levels of depressive symptoms. For the number of pain locations, we found that about 28% reported 0 pain locations, 27% reported 1 pain location, 29% reported 2 pain locations, and 16% reported 3 pain locations. A total of 18% of the study population have some type of comorbidity including one or more pain locations and medium levels of depressive symptoms or higher. There were consistent patterns indicating that individuals who reported higher numbers of musculoskeletal pain sites also reported higher levels of depressive symptoms. This pattern was confirmed to be non-random as indicated by a significant chi-square test X^2^(6, *N* = 6246) = 369.52,*p* < 0.001. The greatest contribution to the overall chi-square was from the cells with zero pain locations and 3 pain locations. When coded as ordinal categorical variables, we also found a significant bivariate correlation (Spearman’s rho = .24, *p* < 0.001).

**Table 1:** The prevalence and percentages of individuals with low, medium and high levels of depressive symptoms across number of musculoskeletal pain locations at baseline with number of cases and incidence rates for long term sickness absence (LTSA)

Pain Location count	low depressive symptoms	medium depressive symptoms	high depressive symptoms	Total
0	91.4 (1598)	5.8 (102)	2.8 (48)	28.0(1748)
cases/incidence rates	166/.22	15/.33	12/.65	194/.24
Chi2 contribution	31.3	72.6	48.9	152.9
1	82.9 (1415)	11.2 (191)	5.9 (100)	27.3(1706)
cases/incidence rates	227/.36	29/.34	21/.52	277/.36
Chi2 contribution	2.6	5.5	4.6	12.6
2	73.7 (1340)	17.1 (311)	9.2 (168)	29.1(1819)
cases/incidence rates	242/.40	69/.56	31/.46	343/.42
Chi2 contribution	7.8	20.2	9.9	37.9
3	62.9 (612)	23.0 (224)	14.1 (137)	15.6(973)
cases/incidence rates	130/.48	45/.47	35/.62	212/.50
Chi2 contribution	33.7	70.0	62.5	166.2
Total	79.5 (4965)	13.3 (828)	7.25 (453)	6246
cases/incidence rates	791/.34	166/.46	105/.55	1070/.36
Chi2 contribution	75.3	168.3	125.9	369.5

X^2^(6, N = 6246) = 369.52, *p* < 0.001

Incidence rates are presented in cases pr 1,000,000 person days

The lowest incidence rate for LTSA was found for those who reported low levels of depressive symptoms and no pain locations (0.22) and the highest incidence rates were found among participants who reported high levels of depressive symptoms and no pain locations (0.65). Participants who reported three pain locations and high levels of depressive symptoms were also found to have relatively high incidence rates (0.62). Overall incidence rates of LTSA increased across levels of symptoms for both pain and depression. This was generally also the case within each stratum of depressive symptoms and pain, but with two exceptions. Among participants with two pain locations, the incidence rates were highest among those with medium level depressive symptoms (0.56), and in the high depressive symptom strata we found that the highest incidence rate was reported among those with no pain locations.

Table 2 shows hazard ratios (HR) and 95% confidence intervals for the associations between the number of pain locations, level of depressive symptoms and the risk of experiencing a spell of LTSA during follow up. The estimates for the crude associations indicate that individuals who report having pain in one or more locations had an increased risk of LTSA compared to those who do not report any pain with HR estimates ranging from 1.52 (1.26 to 1.82) to 2.08 (1.72 to 2.53). There was also a tendency for increasing risk for LTSA for individuals who reported pain in multiple locations. Having a medium or high level of depressive symptoms was also found to be associated with increased risk of LTSA with HR estimates of 1.31 (1.33 to 1.59) and 1.60 (1.30 to 1.96) respectively. HR estimates for both depressive symptoms and pain attenuated somewhat with increasing levels of statistical adjustment. With the exception of medium level of depressive symptoms, all risk factors maintained confidence intervals that did not include unity in both model 1 and model 2.

**Table 2:** Hazard ratios (HR) and 95% confidence intervals for onset of long term sickness absence (LTSA) within follow up period across levels of depressive symptoms and number of pain locations

	Crude		Model 1		Model 2		Model 3	
	HR	95% CI	HR	95% CI	HR	95% CI	HR	95% CI
Number of pain locations								
0	ref		ref		ref		ref	
1	1.52	(1.26 to 1.82)	1.50	(1.24 to 1.80)	1.44	(1.18 to 1.77)	1.57	(1.26 to 1.95)
2	1.79	(1.50 to 2.13)	1.73	(1.45 to 2.07)	1.56	(1.28 to 1.90)	1.65	(1.33 to 2.04)
3	2.08	(1.72 to 2.53)	1.94	(1.58 to 2.37)	1.81	(1.45 to 2.27)	2.00	(1.56 to 2.57)
Depressive symptoms								
low	ref		ref		ref		ref	
medium	1.31	(1.33 to 1.59)	1.17	(0.98 to 1.40)	1.09	(0.89 to 1.33)	1.52	(0.86 to 2.70)
high	1.60	(1.30 to 1.96)	1.36	(1.10 to 1.70)	1.35	(1.06 to 1.72)	3.07	(1.55 to 6.08)
Interaction terms								
1 pain & medium dep							0.52	(0.25 to 1.08)
1 pain & high dep							0.50	(0.22 to 1.16)
2 pain & medium dep							0.86	(0.45 to 1.64)
2 pain & high dep							0.32	(0.14 to 0.71)
3 pain & medium dep							0.61	(0.31 to 1.21)
3 pain & high dep							0.43	(0.19 to 0.96)
Wald(p)/ df	dep:62.7(0.00)/2 pain:27.(0.00)/3			74.(0.00)/5		70.81(0.00)/11		81.79(0.00)/17

Model 1: Number of pain locations and depressive symptoms are included in the same model (mutually adjusted)

Model 2: Number of pain locations and depressive symptoms are mutually adjusted and further adjusted for age, seniority and cohabitation

In model 3, we added multiplicative interaction terms between depressive symptoms and number of pain locations. The interaction terms quantify the deviation from assumptions of additivity of the independent variables. Two of these terms had confidence intervals with did not include unity (2 pain & high dep and 3 pain and high dep).

An adjusted Wald test indicated that the overall interaction effect was significant X^2^(6, *N* = 5251) = 13.61, *p* < 0.034, suggesting that we can reject the null hypothesis that all interaction-terms were simultaneously equal to zero.

Table 3 shows stratified regression models for the association between depressive symptoms and LTSA within each level of pain count with incidence estimates for all combinations of pain and depressive symptoms. High levels of depressive symptoms was associated with a HR of 3.13 (1.59 to 6.21) for participants reporting high levels of depressive symptoms and no musculoskeletal pain. The HR estimates for both medium and high levels of depressive symptoms were substantially weaker and not statistically significant among participants who reported one or more sites of comorbid pain. This was supported both by the non-significant Wald tests and by the confidence intervals for medium and high depressive symptoms that all contained unity.

**Table 3:** Hazard ratios (HR) and 95% confidence intervals for onset of long term sickness absence (LTSA) within follow up period across levels of depressive symptoms stratified by the number of pain locations

	0 pain locations		1 pain locations		2 pain locations		3 pain locations	
Levels of depressive symptoms	HR	95% CI	HR	95% CI	HR	95% CI	HR	95% CI
low	ref		ref		ref		ref	
medium	1.56	(0.87 to 2.78)	0.79	(0.50 to 1.25)	1.30	(0.96 to 1.77)	0.91	(0.62 to 1.32)
high	3.13	(1.59 to 6.21)	1.55	(0.95 to 2.55)	0.97	(0.62 to 1.50)	1.31	(0.87 to 1.98)
Wald(p) for 8 df		20.07(0.01)		12.14(0.15)		8.07(0.09)		12.98(0.11)

Estimates include adjustment for age, seniority and cohabitation and job-group

Table 4 shows stratified regression models for the number of pain locations across levels of depressive symptoms. Among participants with low levels of depressive symptoms there was an increased risk of LTSA associated with reporting one or more pain locations. Reporting one pain location was associated with a HR of 1.58 (1.27 to 1.97), two pain locations was associated with a HR of 1.64 (1.33 to 2.04) and three pain locations was associated with a HR of 2.00 (1.55 to 2.56). The number of pain locations was not associated with LTSA for individuals with medium or high levels of depressive symptoms. This was indicated both by confidence intervals which all included unity, and by non-significant Wald tests for the models.

**Table 4:** Hazard ratios (HR) and 95% confidence intervals for onset of long term sickness absence (LTSA) within follow up period across number of pain locations, stratified by levels of depressive symptoms

	low depression		medium depression		high depression	
Number of pain locations	HR	95% CI	HR	95% CI	HR	95% CI
0	ref		ref		ref	
1	1.58	(1.27 to 1.97)	0.83	(0.41 to 1.66)	0.80	(0.36 to 1.76)
2	1.64	(1.33 to 2.04)	1.45	(0.79 to 2.67)	0.56	(0.26 to 1.20)
3	2.00	(1.55 to 2.56)	1.26	(0.66 to 2.40)	0.94	(0.44 to 2.00)
Wald(p) for 6 df		54.6(0.00)		10.20(0.33)		14.5(0.11)

Estimates include adjustment for age, seniority and cohabitation and job-group

### Sensitivity analyses

We replicated model 2 and model 3 including additional controls for smoking (current smoker, or not), leisure time physical activity (four levels included as dummy variables) and physical work demands (a continuous measure assessing the cumulative physical work from very, very light to very, very strenuous). These were not included in the main models as they could potentially be mediators for the associations between both pain and depression and LTSA. The inclusion of these variables did not change the estimates nor confidence intervals in any substantial manner relative to those reported in the main results.

## Discussion

The main findings of the current study suggest that associations between depressive symptoms and musculoskeletal pain with LTSA are moderated by the presence of comorbid symptoms. High depressive symptoms and reporting one or more pain locations were associated with increased risk for LTSA among individuals who reported no comorbid symptoms. Neither depressive symptoms nor the number of musculoskeletal pain locations were associated with LTSA among individuals who reported comorbid symptoms. Relatively higher symptom levels of both pain and musculoskeletal pain were found to be associated with higher probability of comorbid symptoms.

The identification of comorbidity between musculoskeletal pain and depressive symptoms was expected as this is widely reported [9, 23, 24]. While many studies utilize location specific indicators of musculoskeletal-pain [25–28], a few other studies also confirm the existence of comorbidity when using the number of pain locations as an indicator of the “widespreadness” of pain [29–31]. In total, we found that 18% of the study population reported some combination of medium to high levels of depressive symptoms and one or more pain locations. Among participants who reported one or more pain locations, *we found that about 18% reported medium levels of depressive symptoms and about 10% reported high levels of depressive symptoms*. For individuals who reported medium to high levels of depressive symptoms we found that about 88% of these reported one or more pain locations. Thus, it appears that while it is common for health care workers to experience musculoskeletal pain without having depressive symptoms, it is less common to experience depressive symptoms without also having musculoskeletal pain.

The statistically significant interaction suggests that the association of depressive symptoms and the number of pain locations with risk of LTSA is not consistent across levels of comorbid symptoms. The stratified models revealed that associations between pain and LTSA are strong and consistent for individuals with low levels of depressive symptoms, and that the number of pain locations is not systematically associated with the risk of LTSA for participants with medium or high levels of depressive symptoms. Similarly, reporting high levels of depressive symptoms was strongly associated with the risk of LTSA for individuals with no reported pain locations, but not among individuals with one or more pain locations.

There are multiple potential explanations for our findings. One potential explanation is that the relative change in risk attenuates with secondary/comorbid symptoms. This would imply that there is a greater relative change in risk for LTSA when going from being healthy to having one symptom compared to the increase in risk from having one, to having two symptoms. This explanation appears to be plausible at least for the most extreme groups where there is a large difference in incidence rates between the reference group (no pain locations and low depressive symptoms) and the highest symptom load for each symptom. The relative difference in incidence rates is much smaller, or even negative, when comparing individuals with high symptom levels of one symptom vs having high levels of both depressive symptoms and musculoskeletal pain. There may also be some methodological explanations for our findings. One such explanation is that the high levels of both physical and emotional demands in care work may be too demanding for individuals with both high symptoms of depression and musculoskeletal pain, and may lead to these individuals dropping out of this line of work. This would imply that the individuals in our sample, with the highest symptom load, are a relatively selected robust group who are able to stay in their job despite their high symptoms. It is also possible that some of the individuals with the most severe symptoms are assigned to less physically and psychologically demanding tasks in preventive efforts.

It is important to note that the group with the highest level of risk (high depressive symptoms and no pain locations) is a very small group. Indeed, less than 1% of the total sample reported this combination of symptoms and only about 10% of individuals with high depression levels report no pain locations. This relatively low number of individuals with high symptom levels reduces statistical power, particularly in the medium and high depression strata. Nevertheless, we remain confident in the interpretation of the findings in light of the significant test of interaction and the consistent difference in the size of HR estimates between the strata with low symptom levels (both pain and depression) versus the other strata.

We were only able to identify two studies which simultaneously included depressive symptoms and musculoskeletal pain as risk factors for LTSA or similar outcomes. Emptage and colleagues [10] studied changes in employment status and onset of work disability and found that having simultaneous symptoms of pain and depression was not associated with significantly different outcomes compared to pain level alone. While this corresponds with our findings, it is important to note that depression alone was not consistently associated with the outcomes in their study. In a cross-sectional study based on data from six different European countries, Demyttenaere and colleagues [11] found that a major depressive episode and high levels of painful physical symptoms had additive effects on a work loss days index. The lack of correspondence with our results may be due to a range of factors including their use of retrospective assessment of work-loss days, a sample from six European countries and the inclusion of both men and women in the analyses. Our study add to this very limited literature about how combinations of symptoms potentially constitute qualitatively different risk profiles of work related outcomes relative to studies only including single risk factors.

Our study has a number of notable strengths including a relatively large sample of female healthcare professionals. Thus, our results should generalize well to this specific occupational group. We also utilized register based outcomes with a relatively long follow up which increases the validity of our outcome assessment and removes any risk of common method bias. Limitations of the study include the reliance on questionnaire information for the symptoms of depression rather than clinical assessment. The time frame of the questions is also a limitation as the questions on depressive symptoms refers to the last two weeks while the questions on musculoskeletal pain refer to the last 12 months. Consequently, we cannot be certain whether the symptoms were present at the same time or if their development follows a chronological sequence. The fact that the questionnaire was conducted during work-time also excludes workers who were sick or unable to be at work the time of the survey, which could result in a study population which is healthier than the population we ideally want to make inference to. The lack of men and different work groups in the sample may also be considered a limitation due to limited generalizability of our findings. Finally, the relatively low response rate could also limit the generalizability of the prevalence estimates, in particular.

Importantly, this study only includes symptoms of musculoskeletal pain and depressive symptoms and does not account for all the other disorders which have known comorbidity with depression and musculoskeletal pain. It is likely that the number of individuals without comorbidity would be further reduced if other disorders with known comorbidity with pain and depressive symptoms would be included. This includes a number of disorders including anxiety disorders [30, 32, 33], substance abuse disorders [32], insomnia [34], eating disorders [30, 35], chronic fatigue and gastrointestinal tract disorders [30].

If future studies identify interactions with other symptoms or disorders, then this could imply that traditional variable oriented analyses may not be appropriate for the investigation of risk of LTSA. The reason for this is that testing and interpreting regression models with three or four way interactions is highly complex and may be practically unfeasible in most cases. Rather we would suggest that it may be necessary to assess how different symptoms typically cluster together in a cluster-analysis or latent class/profile type of model and subsequently identify the extent to which these groups of individuals differ in their risk of LTSA.

Our results have implications for both clinical practice and occupational medicine/epidemiology. For clinicians, our findings impliy that it may be appropriate to also screen or assess clients for musculoskeletal pain upon the recognition depression, and for depression upon the recognition of musculoskeletal pain. Our findings also have implications for occupational epidemiology. The reported interaction between musculoskeletal pain and depressive symptoms imply that studies focusing on the association between either one of these risk factors and LTSA may potentially yield misleading results if they ignore or simply control for the other risk factor. This may be illustrated by comparing the estimates from the stratified models in Tables 3 and 4, with the estimates from model 2. In such a comparison, we find that that model 2 underestimate associations between depressive symptoms and LTSA for participants who do not report comorbid pain. We also find that model 2 overestimates the association between musculoskeletal pain and risk of LTSA for individuals with medium or high levels of depressive symptoms.

We suggest that future studies should attempt to replicate our study design to test the validity and generalizability of our findings. We also encourage studies to investigate the role of other potential comorbidities, and the mechanisms of why depressive symptoms and musculoskeletal pain appear to interact in predicting risk of LTSA.

## Conclusions

This investigation found that the associations between depressive symptoms, symptoms of musculoskeletal pain and LTSA differ for individuals with and without comorbid symptoms. The number of pain locations and high levels of depressive symptoms were associated with increased risk of LTSA among individuals with no comorbid symptoms. Neither depressive symptoms nor musculoskeletal pain were associated with LTSA among individuals with comorbid symptoms.

## Abbreviations

DREAM: The national register on social transfer payments (in Denmark)
HR: Hazard ratio
LTSA: Long term sickness absence
MDI: Major Depression Inventory
